# An Exhaustive Search Algorithm to Aid NMR-Based Structure Determination of Rotationally Symmetric Transmembrane Oligomers

**DOI:** 10.1038/s41598-017-17639-w

**Published:** 2017-12-12

**Authors:** Jing Yang, Alessandro Piai, Hong-Bin Shen, James J. Chou

**Affiliations:** 1 Institute of Image Processing and Pattern Recognition, Shanghai Jiao Tong University, and Key Laboratory of System Control and Information Processing, Ministry of Education of China, Shanghai, 200240 China; 2 Department of Biological Chemistry and Molecular Pharmacology, Harvard Medical School, Boston, Massachusetts, 02115 USA; 3 National Center for Protein Science Shanghai, Shanghai Institute of Biochemistry and Cell Biology, Shanghai Science Research Center, Chinese Academy of Sciences, Shanghai, 200031 China

## Abstract

Nuclear magnetic resonance (NMR) has been an important source of structural restraints for solving structures of oligomeric transmembrane domains (TMDs) of cell surface receptors and viral membrane proteins. In NMR studies, oligomers are assembled using inter-protomer distance restraints. But, for oligomers that are higher than dimer, these distance restraints all have two-fold directional ambiguity, and resolving such ambiguity often requires time-consuming trial-and-error calculations using restrained molecular dynamics (MD) with simulated annealing (SA). We report an Exhaustive Search algorithm for Symmetric Oligomer (ExSSO), which can perform near-complete search of the symmetric conformational space in a very short time. In this approach, the predetermined protomer model is subject to full angular and spatial search within the symmetry space. This approach, which can be applied to any rotationally symmetric oligomers, was validated using the structures of the Fas death receptor, the HIV-1 gp41 fusion protein, the influenza proton channel, and the MCU pore. The algorithm is able to generate approximate oligomer solutions quickly as initial inputs for further refinement using the MD/SA method.

## Introduction

Constructing molecular models by satisfying experimentally derived spatial and angular restraints is a general framework for the generation of three-dimensional protein structures by NMR^1^. The most common method for NMR structure calculation is using restrained molecular dynamics (MD) with simulated annealing (SA)^2–6^. In the MD/SA method, structural restraints are implemented as pseudo potentials that drive the dynamics, but such implementation is difficult for ambiguous restraints as they generate potentials with multiple minima. Distance geometry (DG) is another structure calculation method that was very popular in early NMR applications to structural biology^7,8^. This algorithm, however, is sensitive to small uncertainties in the distance matrix. Furthermore, the Bayesian inference has been proposed for NMR structure determination^9^. This method, which derives a probability distribution for the unknown structure, is more computationally challenging. In general, all these methods are not very effective in handling ambiguous restraints.

**Figure 1:**
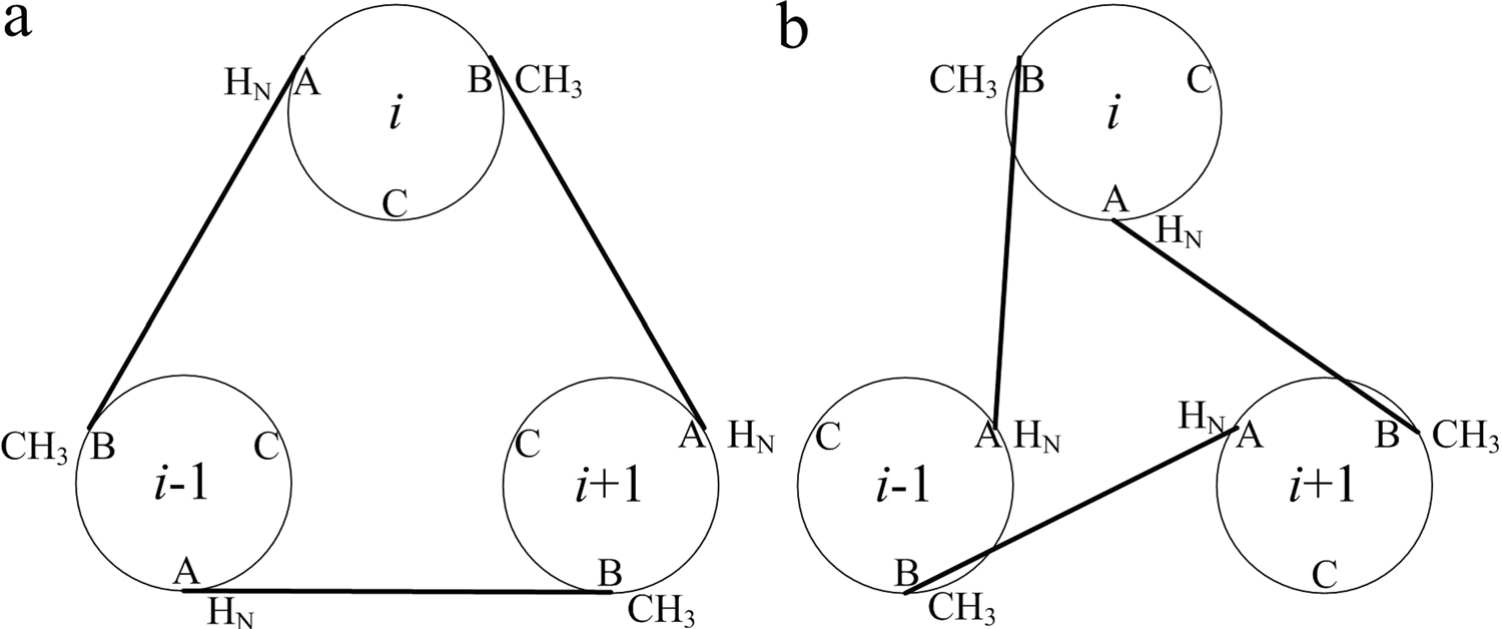
Illustration of the two-fold directional ambiguity of an inter-protomer NOE restraint between the backbone H_N_ and side-chain CH_3_. (**a**) Restraint between H_N_ of residue A in protomer *i* and CH_3_ of residue B in protomer *i* − 1 in counterclockwise arrangement. (**b**) Restraint between H_N_ of residue A in protomer *i* and CH_3_ of residue B in protomer *i* + 1 in clockwise arrangement.

In NMR-based structure determination of transmembrane (TM) oligomers, the key structural restraints are inter-protomer distance restraints derived from nuclear Overhauser enhancement (NOE). These NOEs are typically between the backbone amide proton of one protomer and aliphatic protons of the neighboring protomers^10–13^. For oligomers with *n*-fold rotational symmetry, each NOE restraint between a pair of protomers is duplicated *n* times and assigned respectively to all equivalent pairs of protomers to satisfy the condition of symmetry. In symmetric dimers (*n* = 2), the inter-protomer NOE restraint can be assigned unambiguously between atom *j* of protomer 1 and atom *k* of protomer 2, and between atom *j* of protomer 2 and atom *k* of protomer 1. For *n* ≥ 3, however, each of the NOE-derived restraints has two-fold directional ambiguity. Taking a symmetric trimer as an example, supposing an inter-protomer NOE cross peak between the amide proton of residue A (H_N_(A)) and the methyl proton of residue B (CH_3_(B)) has been identified, it can represent a restraint between H_N_(A) of protomer *i* and CH_3_(B) of protomer *i*-1 (Fig. 1a), or between H_N_(A) of protomer *i*-1 and CH_3_(B) of protomer *i* (Fig. 1b), because the NMR resonances of the protomers are identical. The MD/SA method is suitable for restraints that can be implemented as pseudo potentials, but such potentials cannot be implemented precisely in the case of ambiguous restraints, posing serious problems for energy minimization calculations.

Obviously, algorithms that do not depend on restraint-derived energy landscape are more suitable for resolving ambiguous restraints, and several exhaustive search methods have been proposed previously, including the AmbiPack^14^ and SYMBRANE^15^. These methods are based on the branch-and-bound algorithm to exhaustively search molecular symmetry axis – which is then translated to the oligomer structure – by recursively dividing a cell representing the symmetry space into smaller sub-cells until finding a cell in which the symmetry axes satisfy all the restraints. The search time of this algorithm, however, increases exponentially with fewer restraints because more cells remain, which will be further divided in each of the following iterations of the search. Moreover, it is difficult to implement other restraints such as orientation restraints or the unconventional restraints such as solvent or membrane accessibility^16^.

Inspired by these studies, we sought to develop an exhaustive search algorithm for symmetric oligomers with complexity and searching time unaffected by the amount or form of restraints. The purpose of the program is to allow convenient and fast evaluation of whether the experimental restraints are sufficient to achieve a unique mode of oligomerization. The representative conformations from this program can then be further refined in the standard restrained MD and SA programs.

## Results

**Figure 2:**
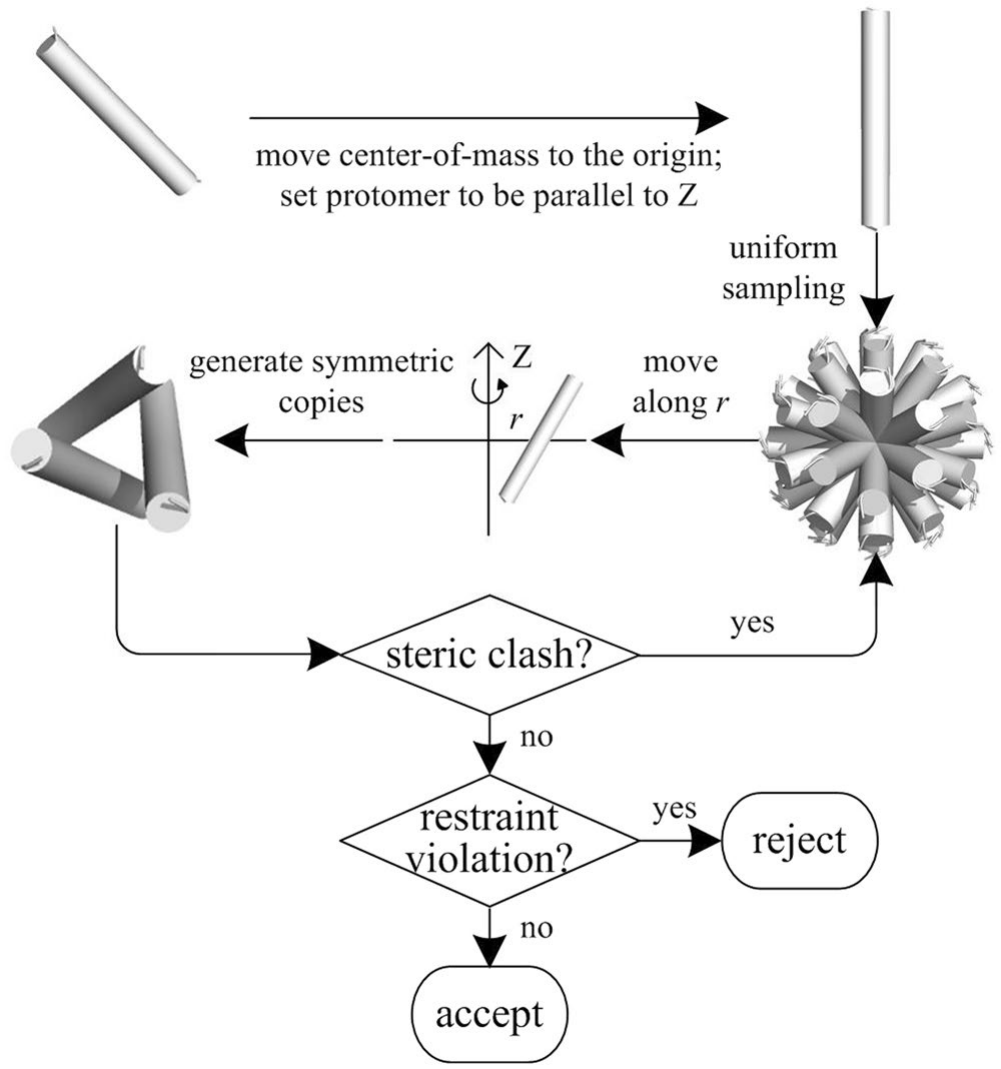
Schematic diagram of the ExSSO algorithm for uniformly searching the conformations of a symmetric oligomer that satisfy experimental restraints. A steric clash is reported when the distance between Cβ atoms from different protomers is less than 3.8 Å (Supplementary Fig. 1).

In the proposed method, named ExSSO and schematically illustrated in Fig. 2, each protomer is treated as a rigid body whose orientation and position relative to the symmetry axis are evaluated. The protomer structure and oligomeric state must be predetermined. In the case of small transmembrane domains (TMDs), e.g., a TM helix, the protomer backbone structure can be initially constructed with the backbone dihedral angles derived from chemical shifts (using, e.g., the TALOS + program^17^). The algorithm assigns the Z-axis as the axis of symmetry and samples the orientation of the protomer by performing an Euler rotation around its center-of-mass with Euler angles α, β, and γ. To ensure near-complete and uniform conformational sampling, we use the following search grid: α = 0 - 2π, Δα = 5°; β = 0 - π/2, Δβ = 5°; γ = 0 - 2π, Δγ = 5°/sin(β). Subsequently, the oriented protomer is placed at distance *r* between the Z-axis and its center-of-mass. By default, the distance *r* is set to the range 3–15 Å and the step size Δ*r* = 0.5 Å is used, because these values were found optimal for the sizes of most TM oligomers investigated by NMR. Moreover, the user is given the option to adjust these settings if needed. For each configuration of the protomer, the oligomer structure is then constructed by generating symmetric copies of the protomer around the Z-axis using the rotational symmetry operator. Structures with steric clashes, as indicated by inter-protomer distances between Cβ atoms (see Supplementary Fig. 1), are not considered. Finally, each of the oligomer structures is evaluated against the inter-protomer restraints using the following scoring system, which quantifies the agreement between each structural model and inter-protomer restraints as follows:

1 $${\rm{\Delta }}=\sqrt{\frac{1}{N}{\sum }_{i=1}^{N}{\delta }_{i}^{2}},$$

where *N* is the number of restraints and *δ*_*i*_ is the deviation in the model from the *i*th restraint. *δ*_*i*_ is defined as:

2 $${\delta }_{i}=\{\begin{array}{c}d-{D}_{i}-{\sigma }_{i},\,d > {D}_{i}+{\sigma }_{i}\\ d-{D}_{i}+{\sigma }_{i},d < {D}_{i}-{\sigma }_{i}\\ 0,\phantom{\rule{4em}{0ex}}\,\,\,{\rm{otherwise}}\end{array},$$

where *D*_*i*_ and *σ*_*i*_ are the value and uncertainty of the *i*th restraint, respectively, and *d* is the corresponding distance calculated from the structural model. As described above, an inter-protomer NOE restraint has two-fold directional ambiguity (Fig. 1). Hence, only the one that is better satisfied by the model is used to represent that NOE restraint.

The NOE-derived inter-protomer restraints typically involve protein side-chain methyl and aromatic groups, which are usually mobile due to side-chain flexibility. To enable the rigid-body conformational search of the protomers without sampling side-chain flexibility, the ExSSO converts each of the NOE restraints to pseudo restraint between the protein backbone heavy atoms including Cα and Cβ. The protons in the NOE restraints are grouped into two groups: (1) those that are close to the backbone (H_N_, Hα, and Hβ), and (2) those that are farther away from the backbone (protons at γ, δ, and ε positions and aromatic protons). The NOE restraints are then classified into three types: (I) between group 1 protons, (II) between group 1 and 2 protons, and (III) between group 2 protons. The type I, II, and III restraints are represented by pseudo restraints between Cα and Cα, between Cα and Cβ, and between Cβ and Cβ, respectively. To assign the proper distance range for the three types of pseudo restraints above, we performed a statistical analysis using 26 membrane protein structures solved by NMR (Supplementary Table 1). For each type (I, II, and III) of observed long-range NOE restraint (inter-protomer or inter-helical), the distance between the two corresponding backbone atoms (Cα, Cβ) was extracted from the structure. Then, by fitting the distribution for each of the three types of distances to Gaussian function (Supplementary Fig. 2), we derived the mean distance and standard deviation for the three restraint types: 6.3 ± 1.7 Å (type I), 6.8 ± 1.5 Å (type II), and 7.3 ± 1.5 Å (type III). For each restraint type, the mean distance was assigned to *D*_*i*_ in Eq. 2 and the standard deviation to the associated uncertainty σ_*i*_.

During the search, the ExSSO keeps a *conformation queue* of representative models with Δ ≤ $\bar{\sigma }$, where $\bar{\sigma }$ is the average restraint uncertainty (~1.5 Å). The use of $\bar{\sigma }$ in collecting the models is based on the argument that the discrepancy between a model and restraints is acceptable if it is within the restraint uncertainty. Initially, the queue is empty. Then, models with Δ ≤ $\bar{\sigma }$ are added to the queue and ranked according to Δ in ascending order. The first model in the queue (or the model with the smallest Δ) is kept by default. Then, starting from the second model in the queue, each model is compared with the other existing models in the queue and is removed from the queue if it is similar to another model with RMSD ≤ 0.5 Å. Lastly, representative models are identified using a clustering algorithm, i.e., only cluster centers in the *conformation queue* are collected as the *conformational ensemble*. In the clustering algorithm^18^, the first cluster center is identified as the model with the largest number of similar models (with RMSD ≤ 1 Å). Once the cluster is found, the models within the cluster are removed. The procedure is then repeated iteratively to find remaining cluster centers until all models in the *conformation queue* are processed.

**Figure 3:**
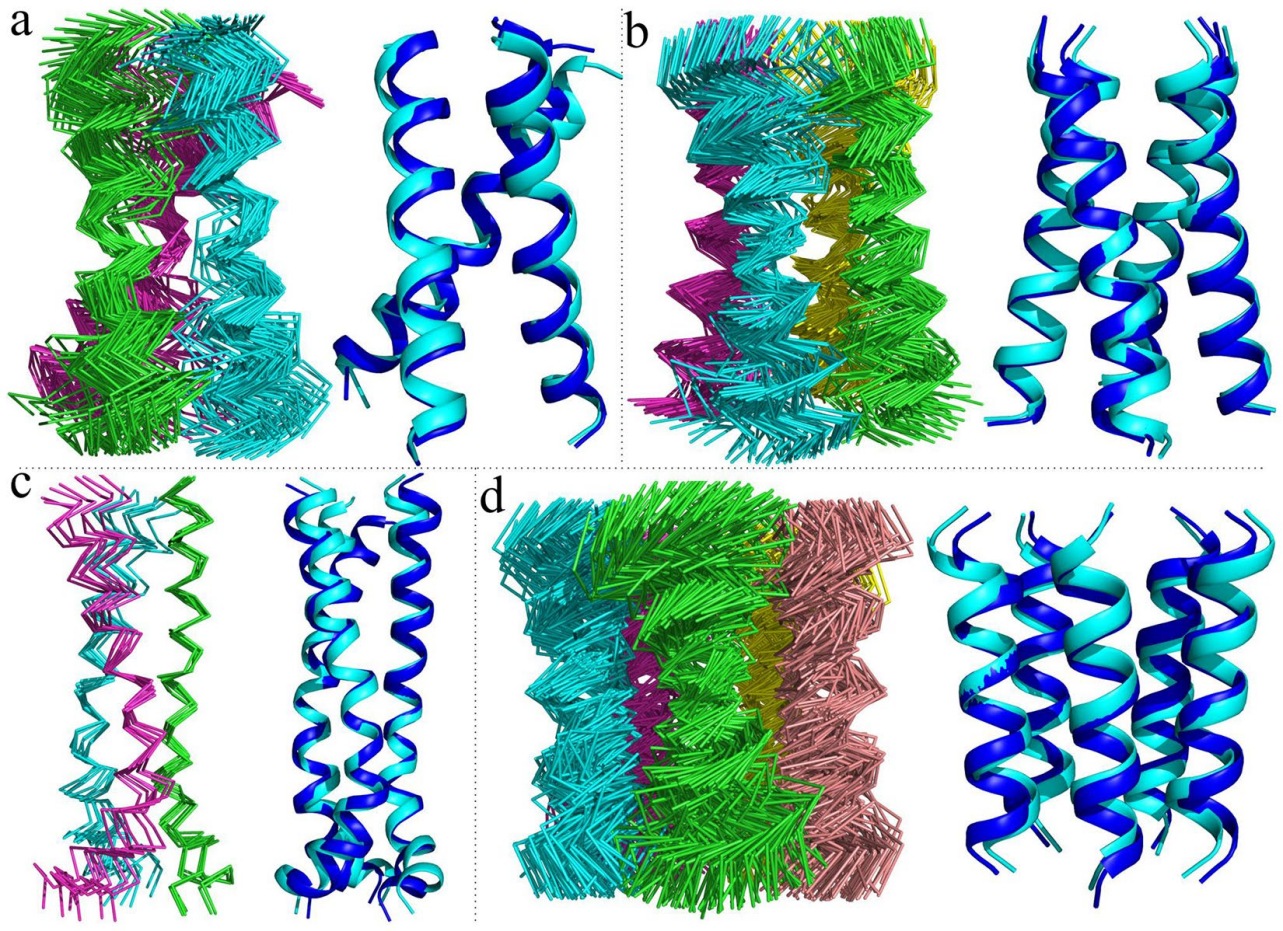
ExSSO-derived *conformational ensemble* and comparison with the known structures. In each sub-figure, the left panel shows the final *conformational ensemble*, and the right panel shows the overlay of the best conformation (cyan) to the known structure (blue). **(a)** The trimeric TMD of the human Fas (PDB ID: 2na7). **(b)** The tetrameric TMD of influenza M2 (PDB ID: 2rlf). **(c)** The trimeric TMD of the HIV-1 gp41 (PDB ID: 5jyn). **(d)** The pentameric complex formed by the second TM helix of *C.elegans* MCU (PDB ID: 5id3).

The above algorithm was tested for several oligomeric TMD structures for which inter-protomer NMR restraints are available: the trimeric TMD of the Fas receptor^19^, the trimeric TMD of the HIV-1 gp41 fusion protein^12^, the tetrameric TMD of influenza M2 channel^20^, and the pentameric TMD of MCU channel pore^21^. The ExSSO parameters used for these applications were: Δα = 5°, Δβ = 5°, Δγ = 5°/sin(β), and Δ*r* = 0.5 Å. The calculations were performed in Mac OS X with a 2.5 GHz Intel Core i5 processor and the results are listed in Table 1 and shown in Fig. 3. The algorithm demonstrated high efficiency as the search time was typically within 20 seconds for each of the four cases (Supplementary Table 2).

**Table 1:** Summary of ExSSO calculation results^a^.

TMD	# of residues	# of restraints^b^	Avg. pairwise RMSD of the ensemble (Å)	RMSD from known structure (Å)^c^
Fas	22	13	4.5	1.5
gp41	34	18	2.8	2.1
M2	23	11	3.8	0.8
MCU	17	9	4.2	1.3

^a^More details in Supplementary Table 2.

^b^NOE-derived inter-protomer restrains.

^c^Backbone Cα difference between the best model (with the smallest ∆ from ExSSO) and the deposited NMR structure.

The *conformational ensembles* (i.e. all the cluster centers found in the *conformation queue*) generated by the ExSSO for each of the four TMDs are displayed in Fig. 3. As can be seen, the spread of the ensemble varied substantially among the four TMDs despite the fact that the average number of inter-protomer restraints per residue is similar for all four cases. Among them, the Fas TMD showed the worst convergence (RMSD = 4.5 Å) (Table 1); it has 62 cluster centers (Fig. 3a), identified from 320 representative models in the *conformation queue*. Notably, although the Fas TMD has more restraints than the M2 TMD, the latter showed better convergence (RMSD = 3.8 Å) (Table 1; Fig. 3b). A careful examination of the restraint list revealed that the inter-protomer restraints of the M2 TMD are better distributed along the TM helix than the Fas TMD. The Fas TMD trimerizes around the central proline, where most of the inter-protomer restraints were found. There are, however, no restraints near the two ends of the TMD, which is consistent with the greater structural divergence observed moving away from the core region of the TMD (Fig. 3a). The HIV-1 gp41 TMD had the best convergence with backbone RMSD of 2.8 Å (Table 1), calculated from 6 cluster centers (Fig. 3c). Finally, the MCU pore TMD also showed mediocre convergence with RMSD of 4.2 Å, calculated from 86 cluster centers (Fig. 3d). Despite the variation in the ensemble spread, the best models (those with the smallest Δ) from ExSSO in the four cases agree remarkably well with the respective known structures, with backbone RMSD from the known models in the range 0.8–2.1 Å (Table 1; Fig. 3).

**Figure 4:**
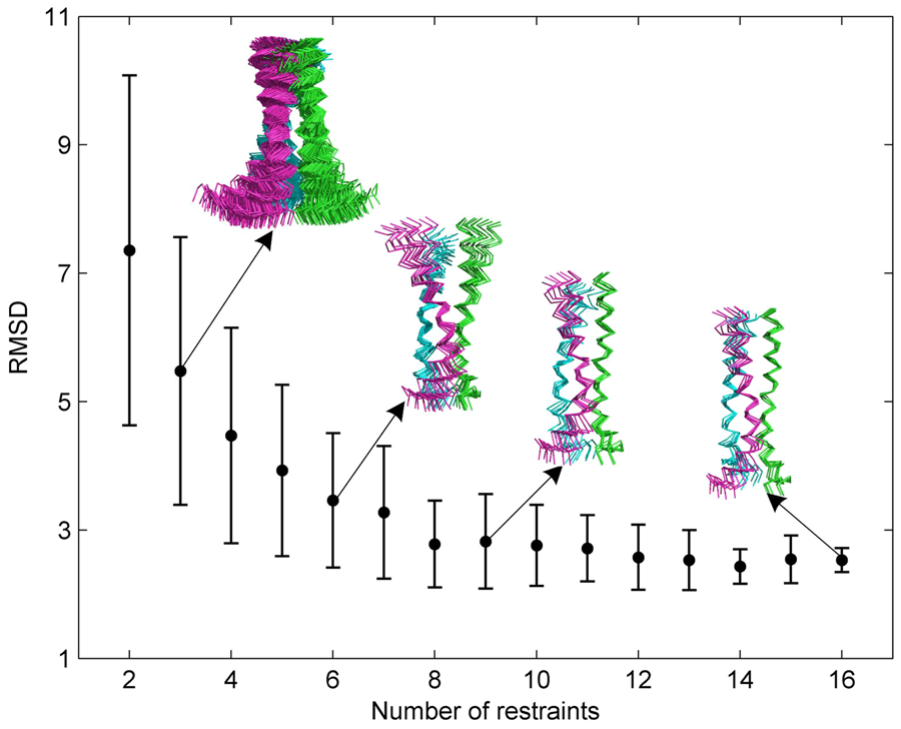
Plot of (*average ensemble RMSD*) vs. (*number of restraints*), showing the structural convergence of the HIV-1 gp41 TMD as a function of the number of inter-protomer restraints used in the ExSSO calculation. The ensemble RMSD is averaged over 100 repetitions and the error bar is the standard deviation (±σ).

We next investigated the influence of reducing the number of restraints on the structural convergence using the TMD of HIV-1 gp41 as a model system. The ExSSO algorithm was tested for different number of inter-protomer restraints, from 16 to 2. For each case, restraints were randomly taken out for an ExSSO calculation and the process was repeated 100 times. The plot of the *average ensemble RMSD* versus the *number of restraints* showed rapid improvement in convergence from 1–5 restraints, but reached steady state at ~9 restraints (Fig. 4). The ensembles generated with 16, 9, 6, and 3 restraints show that the RMSD increased from 2.5 to 5.5 Å with less restraints (Fig. 4). Consistent results were also obtained from performing an identical analysis for the pore-forming TM helix of MCU (Supplementary Fig. 3). In both cases, the plots conform to the fundamental principle of restraint-driven structure determination, and thus further validate the ExSSO algorithm.

## Discussion

In this study, we have developed a fast and efficient algorithm, ExSSO, for uniformly and exhaustively searching for structures of symmetric TM oligomers that satisfy ambiguous and non-ambiguous restraints. We have shown, for several TMDs with known structure and available NMR restraints, that ExSSO can generate not only the highest-score models that agree well with the known structures but also all possible representative models that satisfy the experimental data to within uncertainties.

Historically, exhaustive search has not been commonly used in NMR-based structure determination because it is computationally unrealistic to search the entire conformational space starting from linear polypeptide chains. The search space, however, can be greatly reduced using predetermined secondary structures of the protein. Moreover, in the current application to homo-oligomeric TMD structures, the symmetry constraint further reduces the conformational space, allowing for structure calculation in as little as a few seconds (Supplementary Table 2) on a single 2.5 GHz Intel Core i5 CPU. This type of fast structure turnaround makes the program a useful tool for iterative assignment and resolution of the two-fold ambiguous inter-protomer NOEs during the process of structure determination.

The size of search grid is obviously a central parameter of the algorithm as it has an important consequence on the compromise between the speed and completeness of the search. We found that 5° is an optimal grid size, which produced good results for many cases in a short time. A smaller grid size would take much more time and result in more redundant structures; a larger grid size could miss good models.

The uncertainty in the restraints is another important parameter. In this study, we used a very generous uncertainty (±1.5 Å) to account for side-chain flexibility as well as potentially wrongly assigned restraints (though their number must be much lower than that of the correct restraints). In the case in which a sufficient number of restraints still cannot generate a convergent ensemble, a valid option is to slightly reduce the uncertainties of all the restraints or only of those that are absolutely correct.

To address the tolerance of the ExSSO to inaccurate starting protomer model, we tested the ExSSO calculation using a protomer model of the HIV-1 gp41 TMD that was generated using only the TALOS-derived dihedral restraints. As shown in Supplementary Fig. 4a, this protomer structure is significantly different from the one determined using both TALOS-derived dihedral restraints and local NOE restraints. Despite the substantial deviation in the protomer structure, ExSSO correctly determined the mode of trimer assembly with the inter-protomer NOEs restraints (Supplementary Fig. 4b). This preliminary trimer structure could then be refined to the accurate structure in XPLOR using all NMR restraints (Supplementary Fig. 4c). Therefore, ExSSO proved itself to be an effective tool for generating proximal but correct oligomer models for final refinement using the conventional MD/SA methods.

Finally, the exhaustive conformational search in structure calculation has the obvious advantage of evaluating all ambiguous restraints in a completely unbiased manner, as the search result does not depend on the starting models. The search is also not affected by the complexity introduced by ambiguity in restraints because it systematically evaluates all restraints for all possible conformations. While the current study demonstrates such advantage for ambiguous distance restraints, the exhaustive search approach is in principle generally applicable to all types of structural constraints, including those that are difficult to implement in the form of pseudo potential required by the MD/SA calculation. For example, confinement of TM helices in a lipid bilayer or exclusion of extramembrane domains from the lipid bilayer can be implemented with simple conditional statements to be included in the scoring function (see Supplementary Information). The reported ExSSO program thus represents a versatile framework with which experimental data other than inter-protomer distance restraints can be explored for determining oligomeric TMD structures.

## Method

The calculation modules of the ExSSO program were written in the C++ language. Python scripts were used to operate these modules. All ExSSO calculations were performed in Mac OS X with a 2.5 GHz Intel Core i5 processor. The experimental inter-protomer NOE restraints for the TMDs of the Fas death receptor, the HIV-1 gp41 fusion protein, the influenza M2 channel, and the MCU pore were taken from PDB depositions with PDB IDs 2na7, 5jyn, 2rlf, and 5id3, respectively.

The restrained MD/SA calculation for refining the initial model of the HIV-1 gp41 TMD derived from ExSSO (with inaccurate protomer model) in Supplementary Fig. 4 was performed using the program XPLOR-NIH (version 2.41.1)^5^. In this calculation, the best trimer model from ExSSO (model with the smallest ∆ in Eq. 1) was used as the starting model. The model was refined against the complete set of NMR restraints deposited with PDB ID 5jyn using a SA protocol in which the temperature of the bath was cooled from 1000 to 200 K with steps of 40 K. The NOE restraints were enforced by flat-well harmonic potentials, with the force constant ramped from 2 to 50 kcal/mol Å^−2^ during annealing. Backbone dihedral angle restraints, all with a flat-well (± the corresponding uncertainties) harmonic potential with force constant ramped from 10 to 30 kcal/mol rad^−2^. A total of 50 structures were calculated and 10 lowest energy structures were selected as the final structural ensemble (shown in Supplementary Fig. 4c).

## Electronic supplementary material

Supplementary Information
