# Supplementary material for: An Exhaustive Search Algorithm to Aid NMR-Based Structure Determination of Rotationally Symmetric Transmembrane Oligomers
Source: Sci Rep. 2017 Dec 12;7:17373. doi: 10.1038/s41598-017-17639-w (PMC5727114; doi:10.1038/s41598-017-17639-w)
Supplement: Supplementary file 1 — Supplementary Information [file 41598_2017_17639_MOESM1_ESM.pdf]

An Exhaustive Search Algorithm to Aid NMR-Based Structure Determination of Rotationally Symmetric Transmembrane Oligomers

Jing Yang[1, 2], Alessandro Piai[2], Hong-Bin Shen[1], James J. Chou[2, 3]

[1] Institute of Image Processing and Pattern Recognition, Shanghai Jiao Tong University, and Key Laboratory of System Control and Information Processing, Ministry of Education of China, Shanghai 200240, China

[2] Department of Biological Chemistry and Molecular Pharmacology, Harvard Medical School, Boston, Massachusetts 02115, USA.

[3] National Center for Protein Science Shanghai, Shanghai Institute of Biochemistry and Cell Biology, Shanghai Science Research Center, Chinese Academy of Sciences, Shanghai 200031, China.

Supplementary Table 1. PDB IDs of the 26 membrane proteins used to generate statistics for the three types of inter-protomer NOE restraints.

Supplementary Table 2. More details on the ExSSO calculations.

Supplementary Table 3. PDB IDs of the 103 membrane proteins used to generate statistics for inter-protomer Cβ - Cβ distances in oligomers.

Supplementary Figure 1. The histogram of inter-protomer Cβ - Cβ distances.

Supplementary Figure 2. The histograms of long-range distances for the three types of NOE restraints.

Supplementary Figure 3. Plot of (*average ensemble RMSD*) vs. (*number of restraints*), showing the structural convergence of the pore-forming TM helix of MCU as a function of the number of inter-protomer restraints used in the ExSSO calculation.

Supplementary Figure 4. Effect of protomer structural uncertainty on the ExSSO result.

Example of membrane restraint implementation

The pseudo code of the ExSSO algorithm

Brief usage of the ExSSO program

Supplementary Table 1. PDB IDs of the 26 membrane proteins used to generate statistics for the three types of inter-protomer distance restraints *

1afo	1zll	2hac	2k73	2kih	2kix	2kj1
2knc	2ksy	2kwx	2kyh	2kyv	2l34	2l35
2ljb	2ljc	2ly0	2lzs	2m6x	2n4x	2n9y
2na6	2na7	2rlf	5id3	5jyn		

* The resulting mean and standard deviation of three types of restraints (defined in the main text) are shown in Fig. S2.

Supplementary Table 2. More details on the ExSSO calculations *

TMD	PDB ID	# of models	# of clusters	Search time (s)
Fas	2na7	320	62	11
gp41	5jyn	32	6	19
M2	2rlf	385	58	10
MCU	5id3	1028	86	9

* The ExSSO calculations were performed on Mac OS X with a 2.5 GHz Intel Core i5 processor; the # of models is the number of models in *conformation queue*; the # of clusters is the number of cluster centers stored in the *conformational ensemble*.

Supplementary Table 3. PDB IDs of the 103 membrane proteins used to generate statistics for inter-protomer Cβ - Cβ distances in oligomers *

1afo	1nyj	1xfh	1zll	2bhw	2hac	2j4y
2jwa	2k1	2k1l	2kad	2kdc	2kih	2kix
2kj1	2kpe	2kqt	2kwx	2kyv	2l2t	2l34
2l9u	2lcx	2ljb	2ljc	2ly0	2lzs	2m6i
2m6x	2mic	2mjo	2mpn	2na6	2na7	2nww
2nwx	2rlf	2vt4	2w5j	2wgm	2wie	2wit
2x2v	2zd9	3b5w	3b5y	3b5z	3beh	3bkd
3c9j	3cap	3cn6	3d9s	3dh4	3dww	3e83
3e86	3e89	3e8b	3e8f	3e8g	3e8h	3gjc
3ifx	3j9p	3kbc	3kcu	3kcv	3lbw	3lrb
3lrc	3ob6	3oe9	3vvk	4ain	4c7r	4gpo
4lds	4o6y	4o79	4o7g	4oaa	4p6j	4p6l
4qry	4ri2	4ri3	4rue	4ruf	4tl3	4uc1
4uc2	4uc3	4wol	5duo	5eh4	5etz	5id3
5jef	5jyn	5lv6	5uen	5uk6		

* The resulting statistics are shown in Fig. S1.

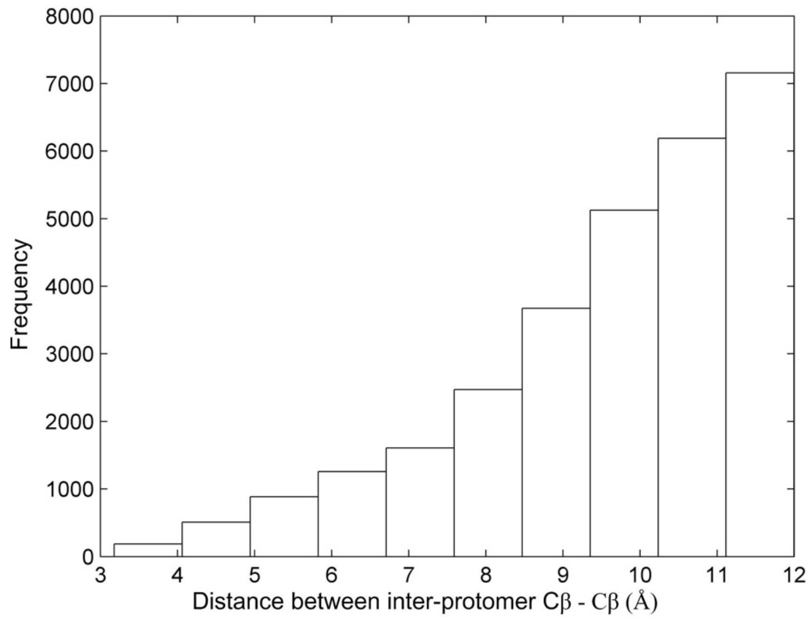

Supplementary Figure 1. The histogram of inter-protomer Cβ - Cβ distances from the structures in Supplementary Table 3. Distances larger than 12 Å were not included in the statistics. The results suggest 3.8 Å is a good value for minimum allowed inter-protomer Cβ - Cβ distances for the ExSSO calculation.

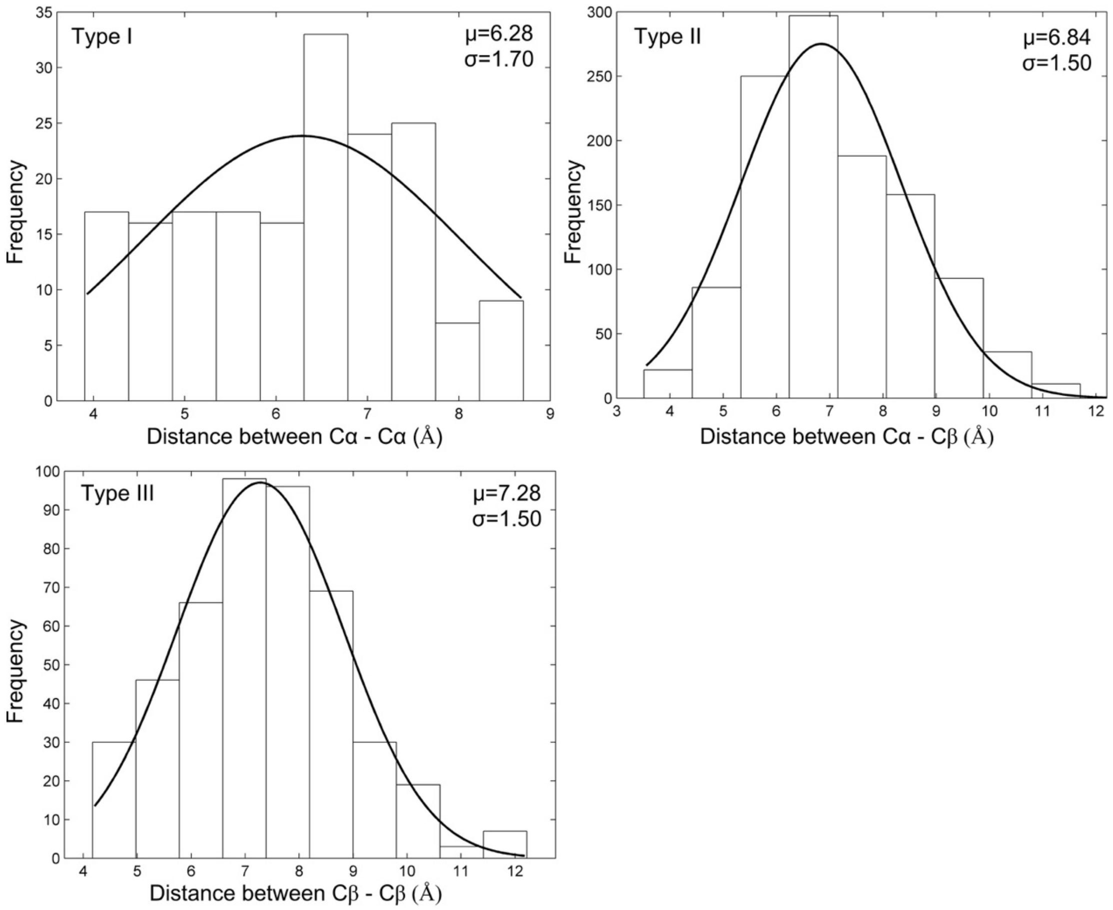

Supplementary Figure 2. The histograms of inter-protomer (or inter-helical) distances for the three types of NOE restraints derived from the 26 membrane protein structures listed in Supplementary Table 1 above. The mean (μ) and standard deviation (σ) for Type I, II, and III distances (defined in the main text) are shown in the corresponding distributions. For the Type I distance distribution, less distances were available for statistics compared with the other types.

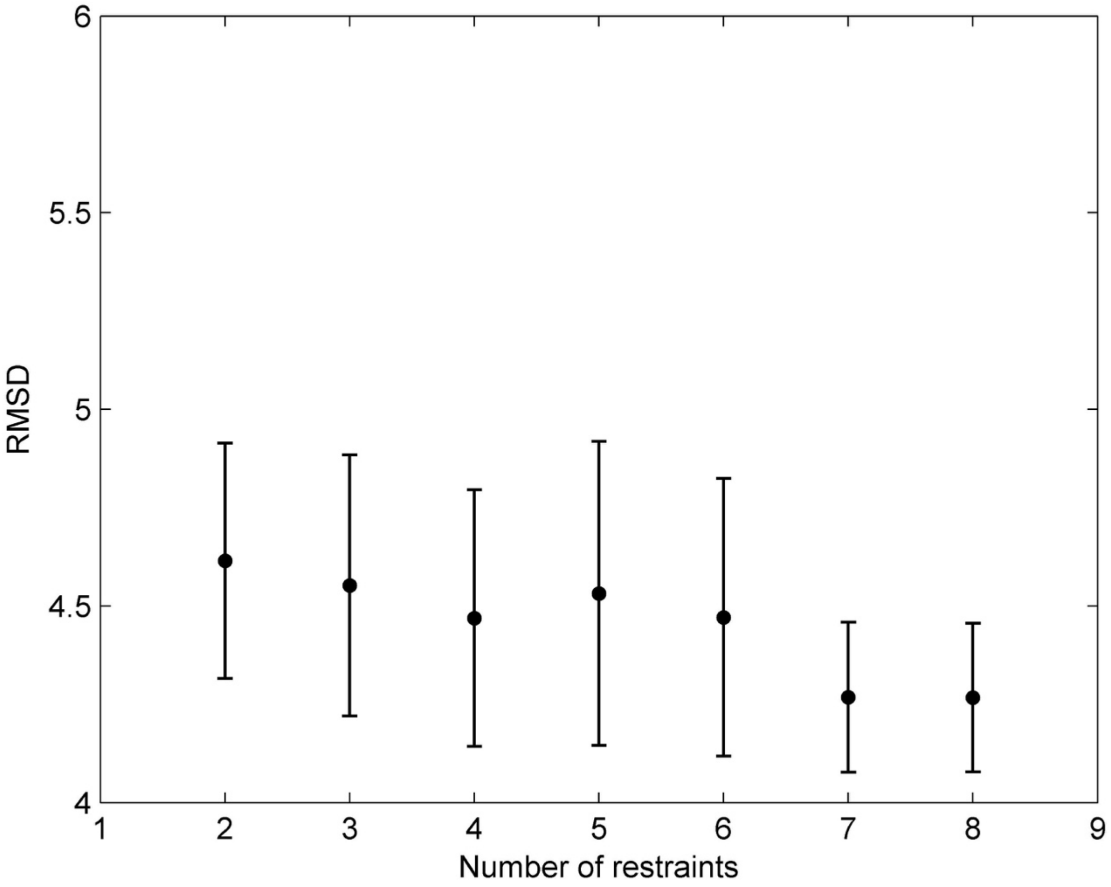

Supplementary Figure 3. Plot of (*average ensemble RMSD*) vs. (*number of restraints*), showing the structural convergence of the pore-forming TM helix of MCU as a function of the number of inter-protomer restraints used in the ExSSO calculation. The ensemble RMSD is averaged over 100 repetitions and the error bar is the standard deviation (±σ).

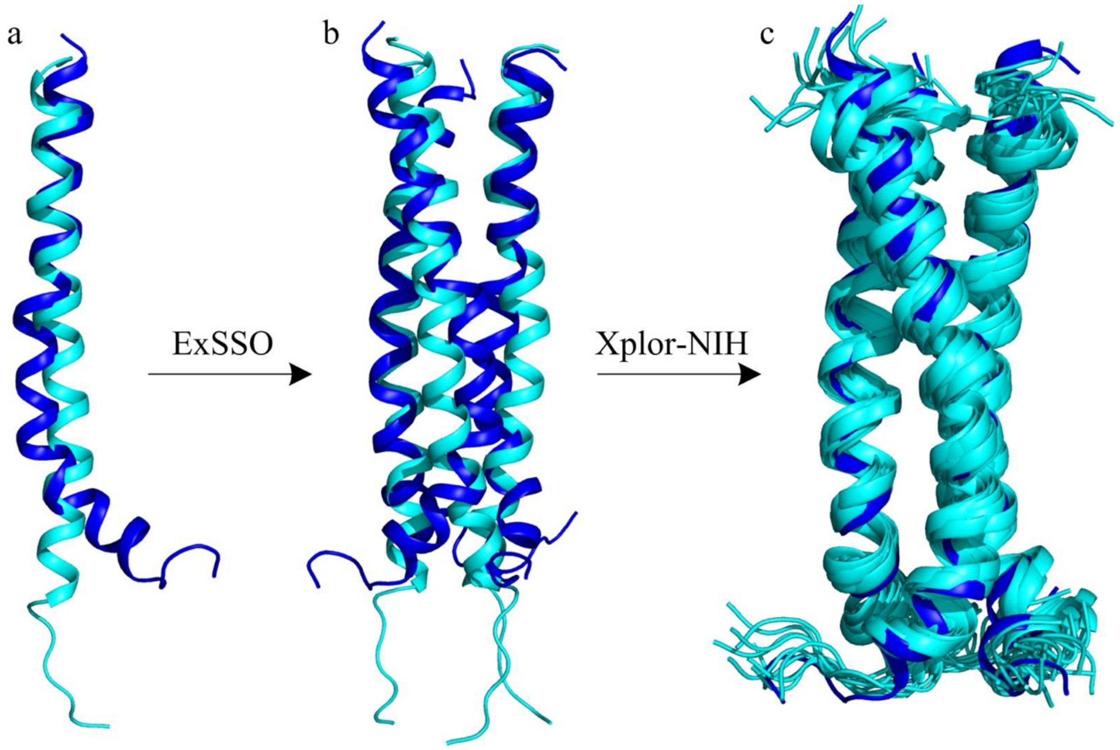

Supplementary Figure 4. Effect of protomer structural uncertainty on the ExSSO result. The ExSSO calculation was performed using a protomer model of the HIV-1 gp41 TMD generated using only the TALOS-derived dihedral restraints. (**a**) Superposition of the protomer model (cyan) generated with only TALOS-derived restraints with the deposited NMR structure (blue). (**b**) Superposition of the best trimer model from ExSSO (cyan) to the deposited NMR structure (blue). (**c**) Superposition of the top 10 structures after further refinement in XPLOR-NIH against all NOE restraints (cyan) with the deposited NMR structure (blue).

Example of membrane restraint implementation

One of the advantages of ExSSO is the convenience of including unconventional restraints such as the TM membrane partition restraints in the exhaustive search. As an example, we have used the HIV-1 gp41 TMD to show how inter-protomer NOEs and membrane restraints can be used together. The membrane partition of gp41 TMD has been proposed based on solvent paramagnetic relaxation enhancement data[1]. In the model, the membrane edges are located between residues 679 and 680 (extracellular boundary) and residues 710 and 711 (intracellular boundary), yielding an estimated bilayer thickness of 48 Å. Therefore, one membrane boundary can be represented by the average coordinates of the N atoms of residues 679 and 680 and the other boundary by the average coordinates of N atoms of residues 710 and 711. The membrane constraint is simply that the distance between the two boundaries, which is the membrane thickness, should be 48 ± 1.5 Å. For each sampled conformation, the structure was rejected if the above condition was false. We found that only 4705 conformations were accepted by using the membrane constraint alone. When integrated with inter-protomer NOE restraints, it helped to reduce the size of the *conformation queue* from 32 to 15, from which 3 cluster centers were obtained (50% less than what was obtained when using inter-protomer NOE restraints alone).

The pseudo code of the ExSSO algorithm

Input:
protomer structure *S*
inter-protomer NOE restraints *R*
number of protomers *N*
Output:
representative models *M*
Pseudo code:

```
function exhaustive_search(S, R, N)
   [α, β, γ] = uniform_sampling()
   for each combination of three Euler angles, do
      for r = 3 : 0.5 : 15
         S1 = rotate_transit(α, β, γ, r)
         S2 = generate_copies(S1, N)
         if (!has_clash(S2) && score_model(S2) ≤ 1.5)
            CQ ← S2
   remove_redundancy(CQ, 0.5)
```

```
   M = density_clustering(CQ, 1.0)
   return M
end

function uniform_sampling()
   for β = 0 : 5π/180 : π/2
      for γ = 0 : 5π/180/sin(β) : 2π
         for α = 0 : 5π/180 : 2π
            return [α, β, γ]
end
```

Brief usage of ExSSO

ExSSO can be downloaded at: http://www.csbio.sjtu.edu.cn/bioinf/ExSSO/.

1. Installation
Download the ExSSO package and unpack it to the directory called ExSSO.
$ tar –xzvf ExSSO_1.0_linux.tar.gz

2. File formats
ExSSO requires at least two input files: the protomer structure file and the inter-protomer restraint file (the membrane constraint file or other new implemented constraint files are optional). The protomer structure file contains the atomic coordinates of the protomer in standard PDB format. The inter-protomer NOE restraints have the following format:

(AA *ResNum1* and ATOM *AtomName1*) (AA *ResNum2* and ATOM *AtomName2*)

where *ResNum* is the residue number in the protomer structure and *AtomName* must be HN, HA, HB, HG, HD, HE, HZ or HH.

The membrane restraint has the following format:

(AA *ResNum1* and *ResNum2*) and (AA *ResNum3* and *ResNum4*) *Distance*

where *ResNum1* and *ResNum2* represent the location of one membrane boundary, *ResNum3* and *ResNum4* represent the location of the other membrane boundary, and *Distance* is the estimated thickness of lipid bilayer.

All examples can be found in the ExSSO package (ExSSO/example).

3. Parameter setting
Open configure.py in the ExSSO/script folder and set parameters including protein name, home directory of ExSSO, file path of the protomer structure, file path of NOE restraints and the number of protomers, users can also change default parameters.

```
################# Input parameter setting ##################
# Protein name
proteinName = 'HIVtmd'

# Home directory of ExSSO
homeDir = '/home/user/ExSSO'

# Protomer structure
pdbFile = '/home/user/ExSSO/example/HIV/protomer.pdb'

# Inter-protomer NOE restraints
noeFile = '/home/user/ExSSO/example/HIV/restraints.noe'

# Membrane restraint (optional)
memFile = '/home/user/ExSSO/example/HIV/restraints.mem'

# Number of protomers
numProtomer = 3

# Use membrane restraint, 1 for “yes” and 0 for “no”
memResFlag = 1

################# Search parameter setting #################
# Step size for Euler angles
ss_angle = 5

# Distance between Z-axis (the symmetry axis) and center-of-mass
# Begin with 3.0
r_beg = 3.0
# End up with 15.0
r_end = 15.0

# Step size for distance r
ss_r = 0.5

# The average restraint uncertainty used for the ACCEPT criterion (e.g., if big
delta in Eq. 1 =< uncertainty, accept the model).
uncertainty = 1.5 (in angstrom)
###################### End of setting ######################
```

4. Running the script

a. In the command line, type the following:

```
$ python configure.py
```

A file called *param* will be created in ../HIVtmd/, which contains all parameters of ExSSO.

b. To check the format of protomer structure file and restraint file, run the following script:

$ python check_format.py -p ../HIVtmd/param -t pdb
for checking the PDB file only

$ python check_format.py -p ../HIVtmd/param -t noe
for checking the NOE restraint file only

$ python check_format.py -p ../HIVtmd/param -t mem
for checking the membrane restraint file only

$ python check_format.py -p ../HIVtmd/param -t all
for checking all prerequisite input files

c. To search conformations, run the following script:

$ python exhaustive_search.py -p ../HIVtmd/param

Representative conformations (cluster centers; see manuscript for more description) are stored in ../HIVtmd/HIVtmd.cs

d. To generate the final structures of symmetric oligomer, which can be fed into Xplor-NIH for further refinement, run the following script:

$ python get_structure.py -p ../HIVtmd/param

All cluster center structures (HIVtmd_*.pdb) can be found in ../HIVtmd/. The structures are sorted from the best to the worst.

Reference

1 Dev, J. *et al.* Structural basis for membrane anchoring of HIV-1 envelope spike. *Science* **353**, 172-175 (2016).
